# Supplementary figures and images for: Alzheimer’s disease protective allele of Clusterin modulates neuronal excitability through lipid-droplet-mediated neuron-glia communication
Source: Mol Neurodegener. 2025 May 3;20:51. doi: 10.1186/s13024-025-00840-1 (PMC12049787; doi:10.1186/s13024-025-00840-1)

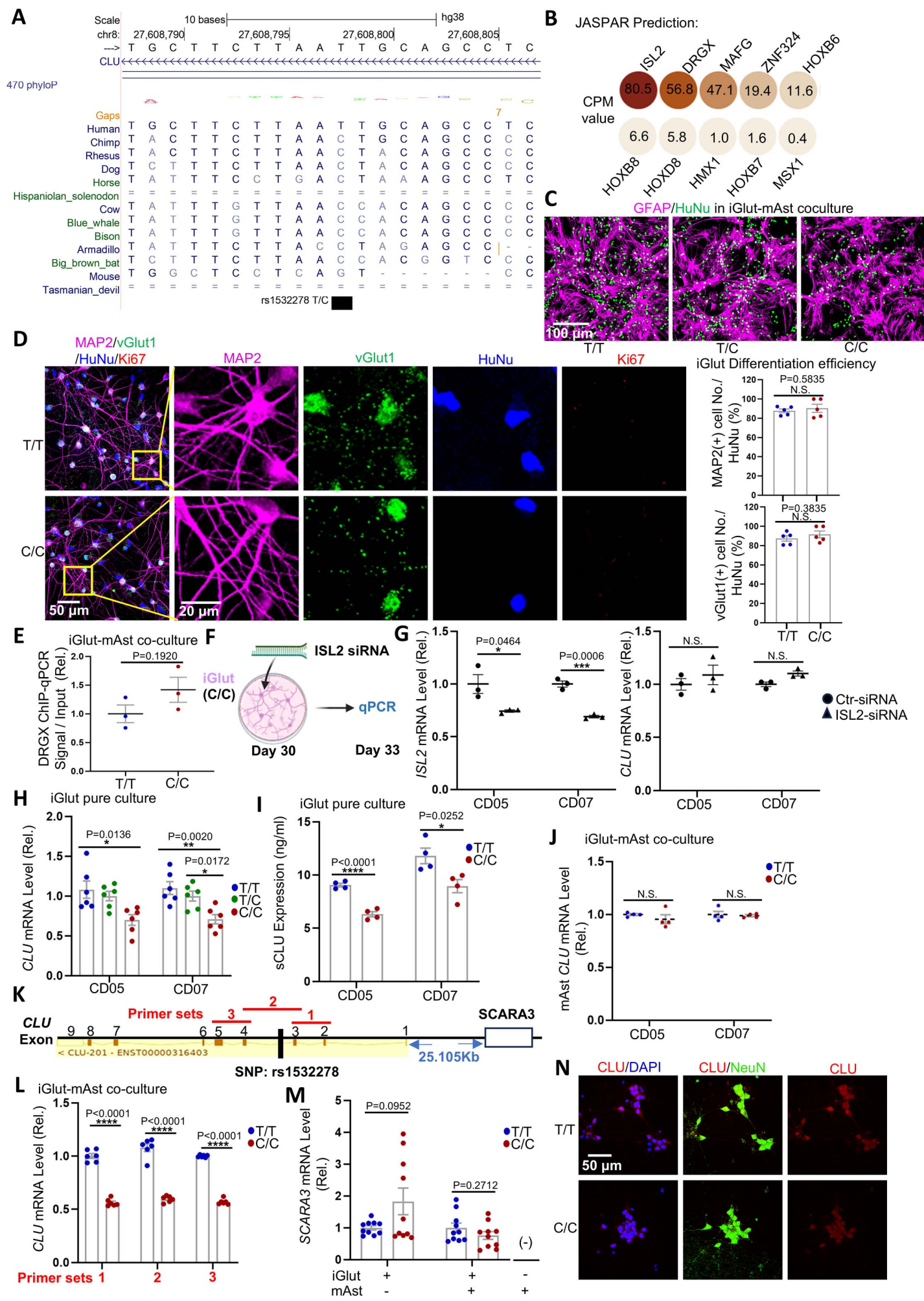

Supplement: Supplementary file 1 — Additional file 1. Figure S1: Bioinformatic and experimental validation of the regulatory effect of rs1532278 on TF-binding and CLU expression, related to Fig. 1. (A) Multiz alignment and phyloP conservation (470 mammals) around rs1532278 (from UCSC hg38 genome browser). (B) JASPAR predicted TF binding sites at rs1532278 and TF expression levels in iGlut (10/23 predicted TFs can be detected by RNA seq). CPM, counts per million reads. (C) Representative images (CD07 line) of iGlut of all three genotypes are also shown, related to Fig. 1D (bottom panel); GFAP and HuNu (human nuclear antigen) staining shows specificity of HuNu and MAP2 staining for iGlut in iGlut-mAst co-cultures. (D) No difference of iGlut differentiation efficiency were found between T/T and CC carriers in iGlut-mAst co-cultures, and none proliferating cells were observed in these neurons indicated by negative staining of Ki67. HuNu +, human cells. n=5 coverslips per group from one differentiation of both CD05 and CD07 lines (2-3 clones per line, one coverslip per clone and 4-5 images per coverslip). (E) DRGX ChIP-qPCR for iGlut-mAst co-cultures of CD07 line on day 30. n=3 biological replicates per group (one clone with 3 biological replicates from the CD07 line) from one independent differentiation. (F-G) ISL2 siRNA knockdown in day-30 pure iGlut (C/C) cultures. Samples of 72 hours post-siRNA transfection were used for qPCR. n=3 biological replicates from one clone per line in one independent differentiation. (H) CLU mRNA levels in iGlut pure cultures. n=6 biological replicates per group (2-3 clones per line and 2-3 biological replicates for each clone) from two independent differentiations of each line (I) sCLU levels detected by ELISA from the supernatant of iGlut pure cultures. n=4 biological replicates per group (2 clones per line and 2 biological replicates for each clone) from two independent differentiations of each line. (J) CLU mRNA levels of mAst in iGlut-mAst co-cultures. n=4 biological r [file 13024_2025_840_MOESM1_ESM.zip › Figure S1.pdf]

Fig 2E

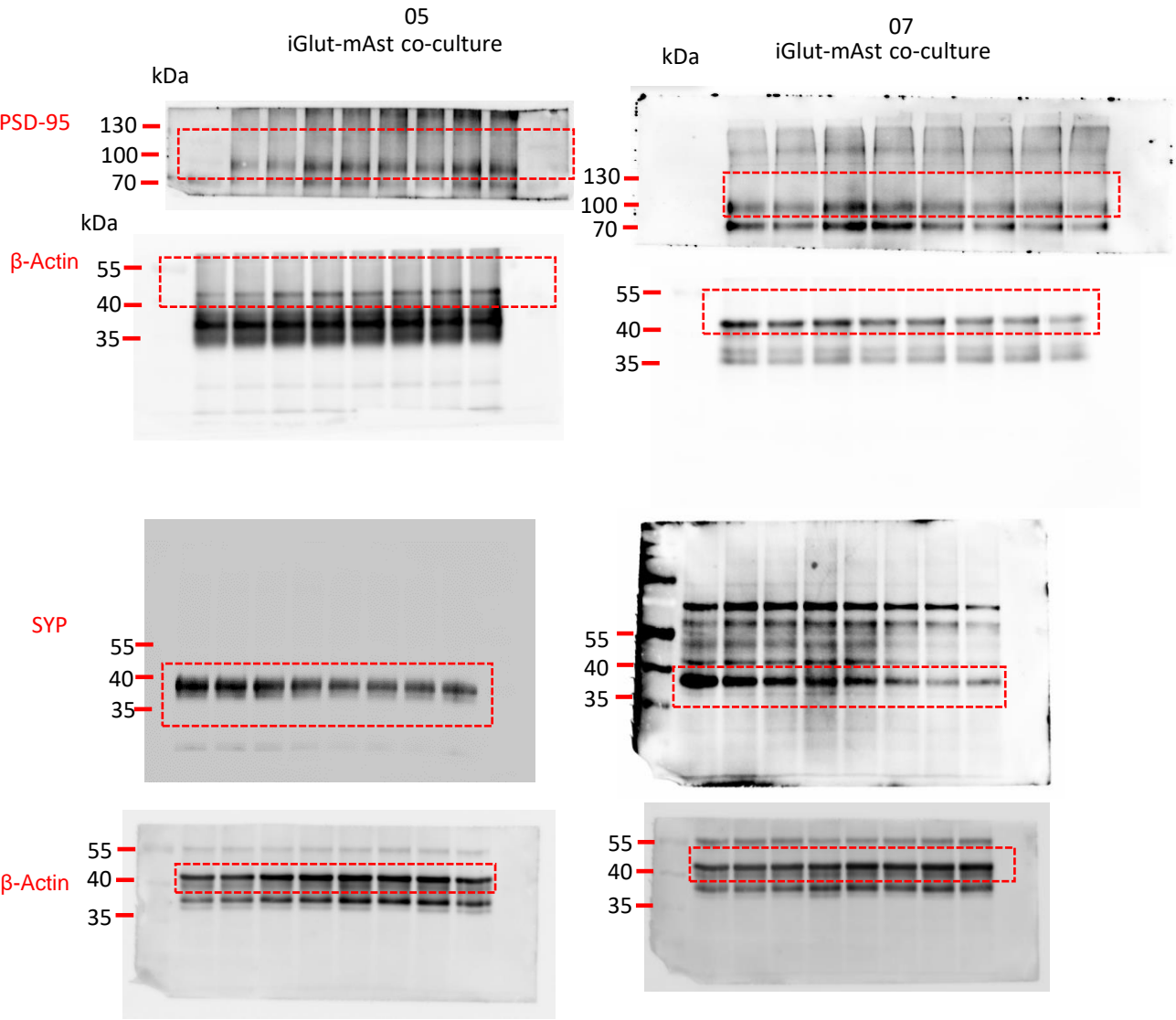

Fig S3B

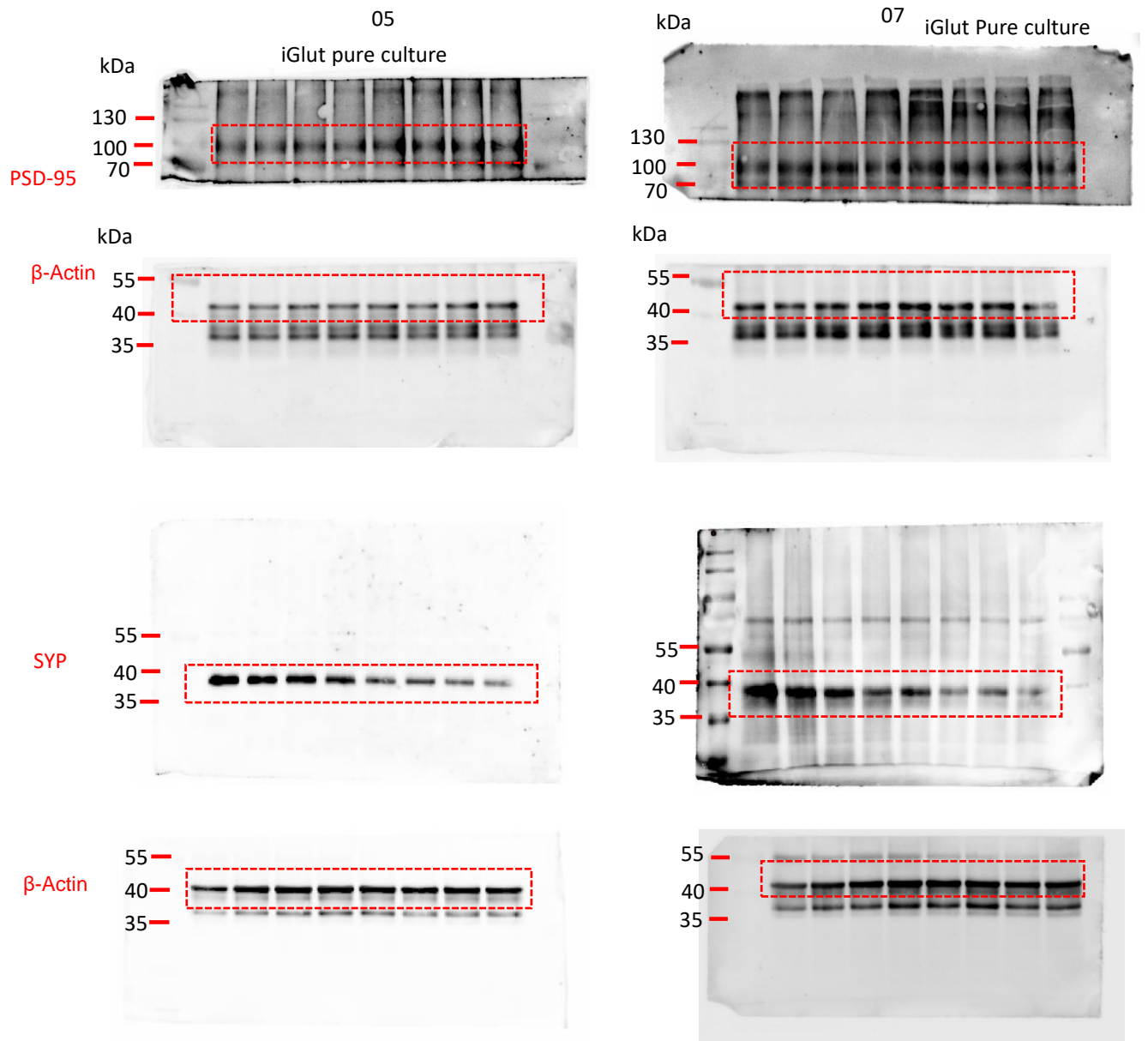

Fig 3C

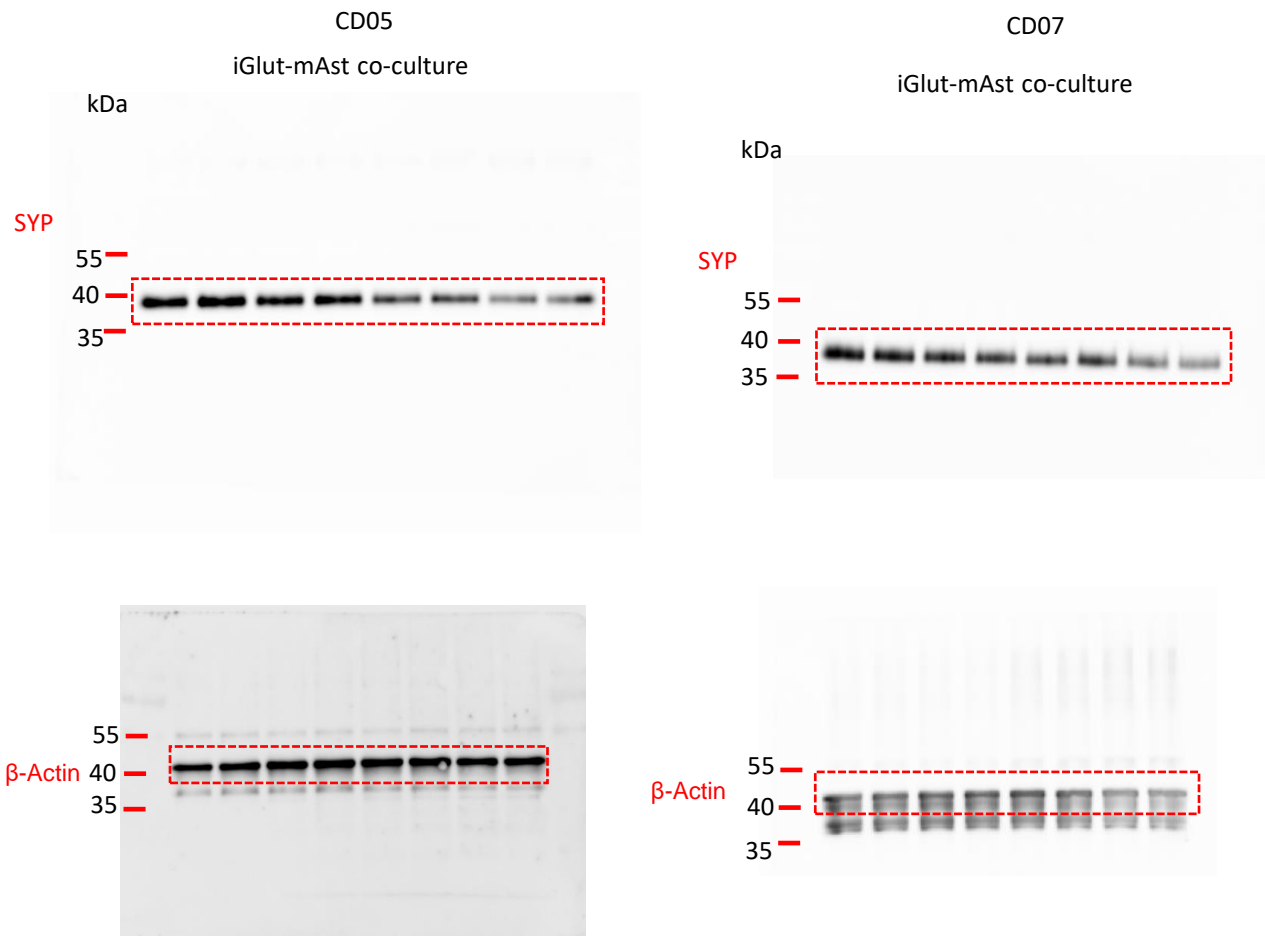

Fig S4E

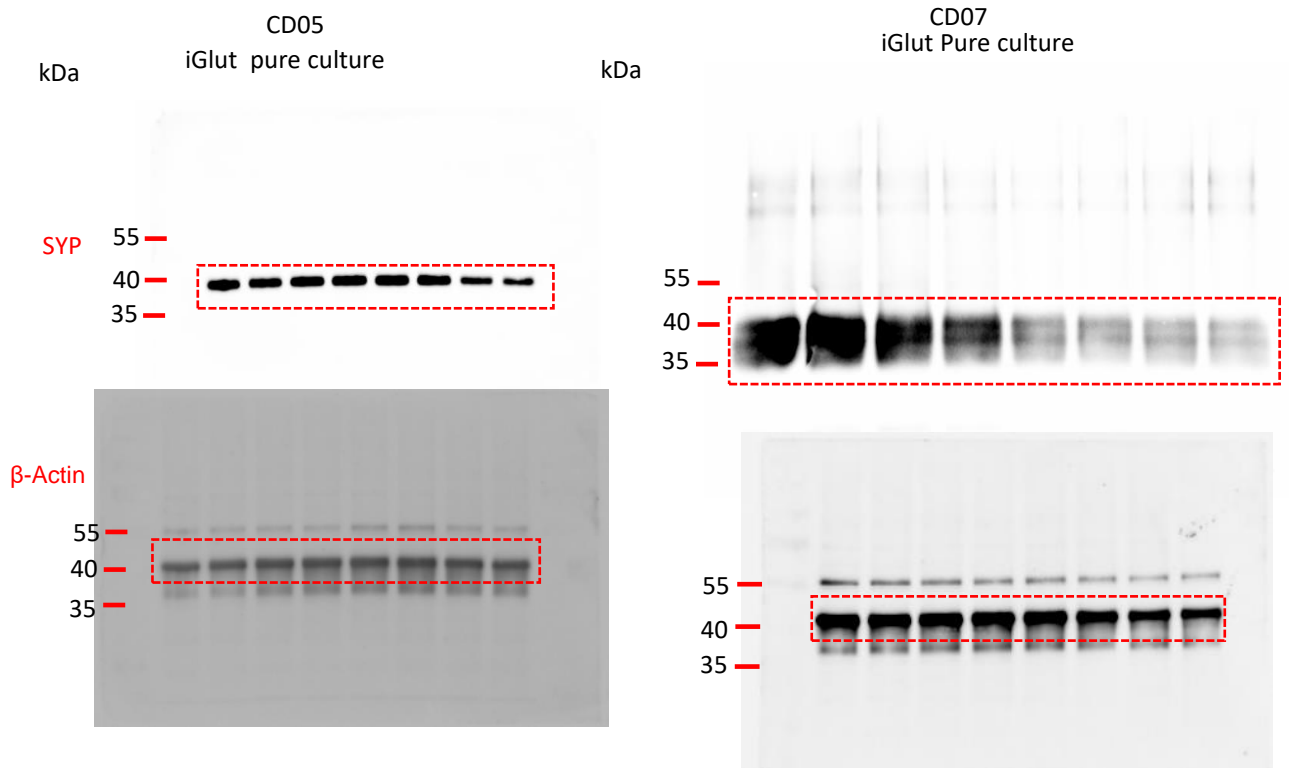

Supplement: Supplementary file 1 — Additional file 1. Figure S1: Bioinformatic and experimental validation of the regulatory effect of rs1532278 on TF-binding and CLU expression, related to Fig. 1. (A) Multiz alignment and phyloP conservation (470 mammals) around rs1532278 (from UCSC hg38 genome browser). (B) JASPAR predicted TF binding sites at rs1532278 and TF expression levels in iGlut (10/23 predicted TFs can be detected by RNA seq). CPM, counts per million reads. (C) Representative images (CD07 line) of iGlut of all three genotypes are also shown, related to Fig. 1D (bottom panel); GFAP and HuNu (human nuclear antigen) staining shows specificity of HuNu and MAP2 staining for iGlut in iGlut-mAst co-cultures. (D) No difference of iGlut differentiation efficiency were found between T/T and CC carriers in iGlut-mAst co-cultures, and none proliferating cells were observed in these neurons indicated by negative staining of Ki67. HuNu +, human cells. n=5 coverslips per group from one differentiation of both CD05 and CD07 lines (2-3 clones per line, one coverslip per clone and 4-5 images per coverslip). (E) DRGX ChIP-qPCR for iGlut-mAst co-cultures of CD07 line on day 30. n=3 biological replicates per group (one clone with 3 biological replicates from the CD07 line) from one independent differentiation. (F-G) ISL2 siRNA knockdown in day-30 pure iGlut (C/C) cultures. Samples of 72 hours post-siRNA transfection were used for qPCR. n=3 biological replicates from one clone per line in one independent differentiation. (H) CLU mRNA levels in iGlut pure cultures. n=6 biological replicates per group (2-3 clones per line and 2-3 biological replicates for each clone) from two independent differentiations of each line (I) sCLU levels detected by ELISA from the supernatant of iGlut pure cultures. n=4 biological replicates per group (2 clones per line and 2 biological replicates for each clone) from two independent differentiations of each line. (J) CLU mRNA levels of mAst in iGlut-mAst co-cultures. n=4 biological r [file 13024_2025_840_MOESM1_ESM.zip › figure S10.pdf]

**A**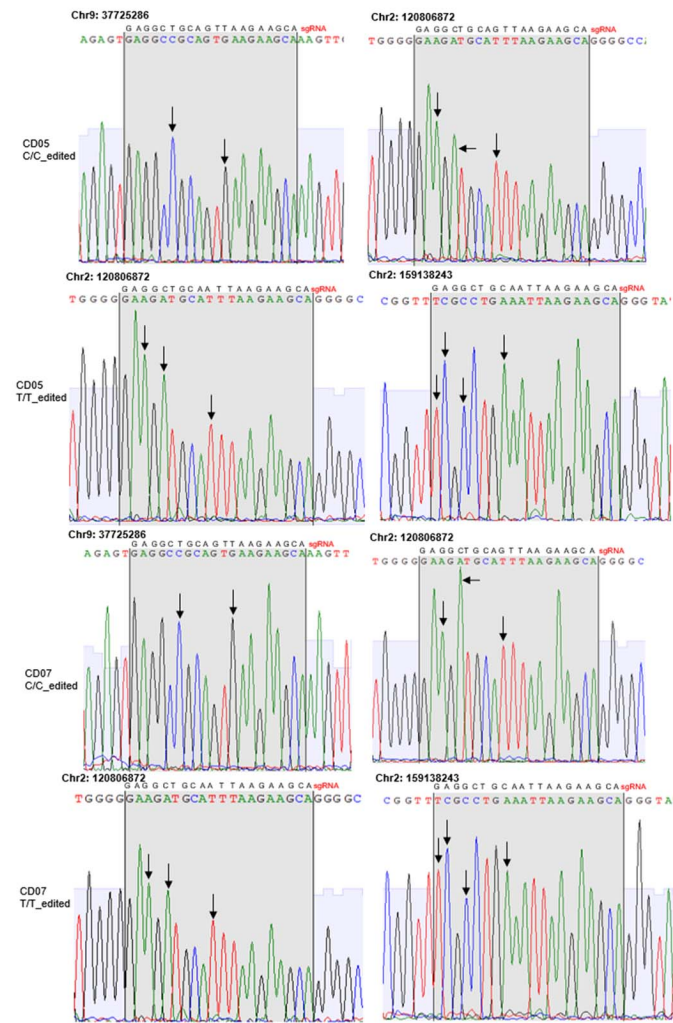**B**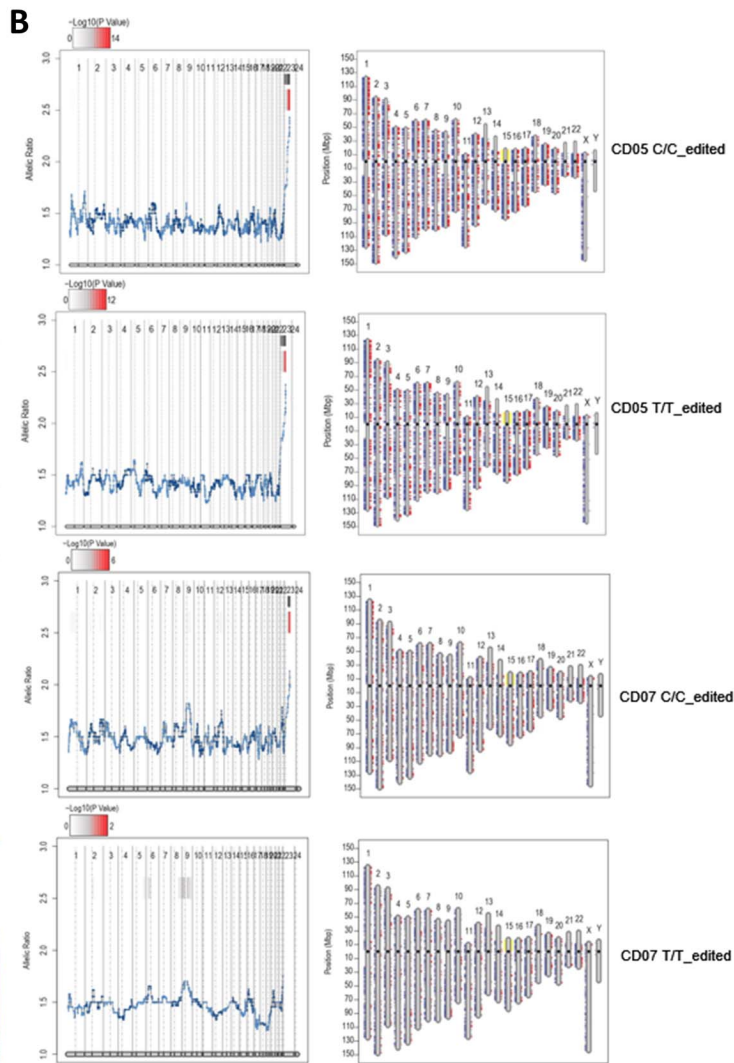**C**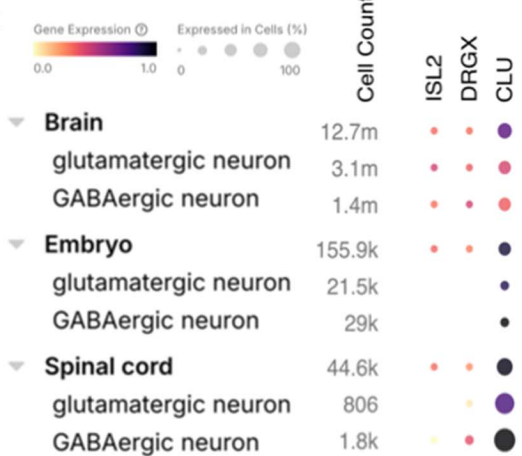**D**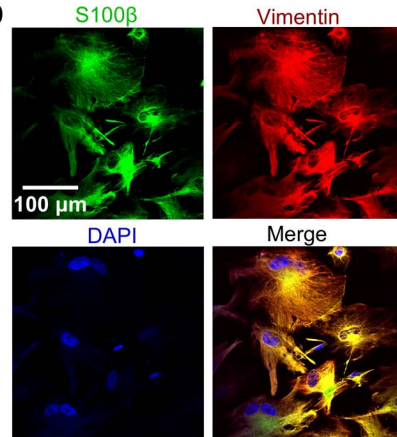**E**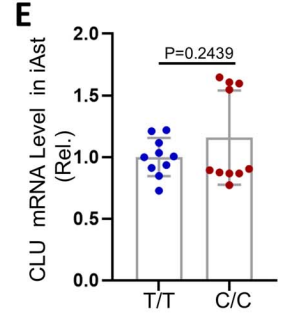

Supplement: Supplementary file 1 — Additional file 1. Figure S1: Bioinformatic and experimental validation of the regulatory effect of rs1532278 on TF-binding and CLU expression, related to Fig. 1. (A) Multiz alignment and phyloP conservation (470 mammals) around rs1532278 (from UCSC hg38 genome browser). (B) JASPAR predicted TF binding sites at rs1532278 and TF expression levels in iGlut (10/23 predicted TFs can be detected by RNA seq). CPM, counts per million reads. (C) Representative images (CD07 line) of iGlut of all three genotypes are also shown, related to Fig. 1D (bottom panel); GFAP and HuNu (human nuclear antigen) staining shows specificity of HuNu and MAP2 staining for iGlut in iGlut-mAst co-cultures. (D) No difference of iGlut differentiation efficiency were found between T/T and CC carriers in iGlut-mAst co-cultures, and none proliferating cells were observed in these neurons indicated by negative staining of Ki67. HuNu +, human cells. n=5 coverslips per group from one differentiation of both CD05 and CD07 lines (2-3 clones per line, one coverslip per clone and 4-5 images per coverslip). (E) DRGX ChIP-qPCR for iGlut-mAst co-cultures of CD07 line on day 30. n=3 biological replicates per group (one clone with 3 biological replicates from the CD07 line) from one independent differentiation. (F-G) ISL2 siRNA knockdown in day-30 pure iGlut (C/C) cultures. Samples of 72 hours post-siRNA transfection were used for qPCR. n=3 biological replicates from one clone per line in one independent differentiation. (H) CLU mRNA levels in iGlut pure cultures. n=6 biological replicates per group (2-3 clones per line and 2-3 biological replicates for each clone) from two independent differentiations of each line (I) sCLU levels detected by ELISA from the supernatant of iGlut pure cultures. n=4 biological replicates per group (2 clones per line and 2 biological replicates for each clone) from two independent differentiations of each line. (J) CLU mRNA levels of mAst in iGlut-mAst co-cultures. n=4 biological r [file 13024_2025_840_MOESM1_ESM.zip › figure S2.pdf]

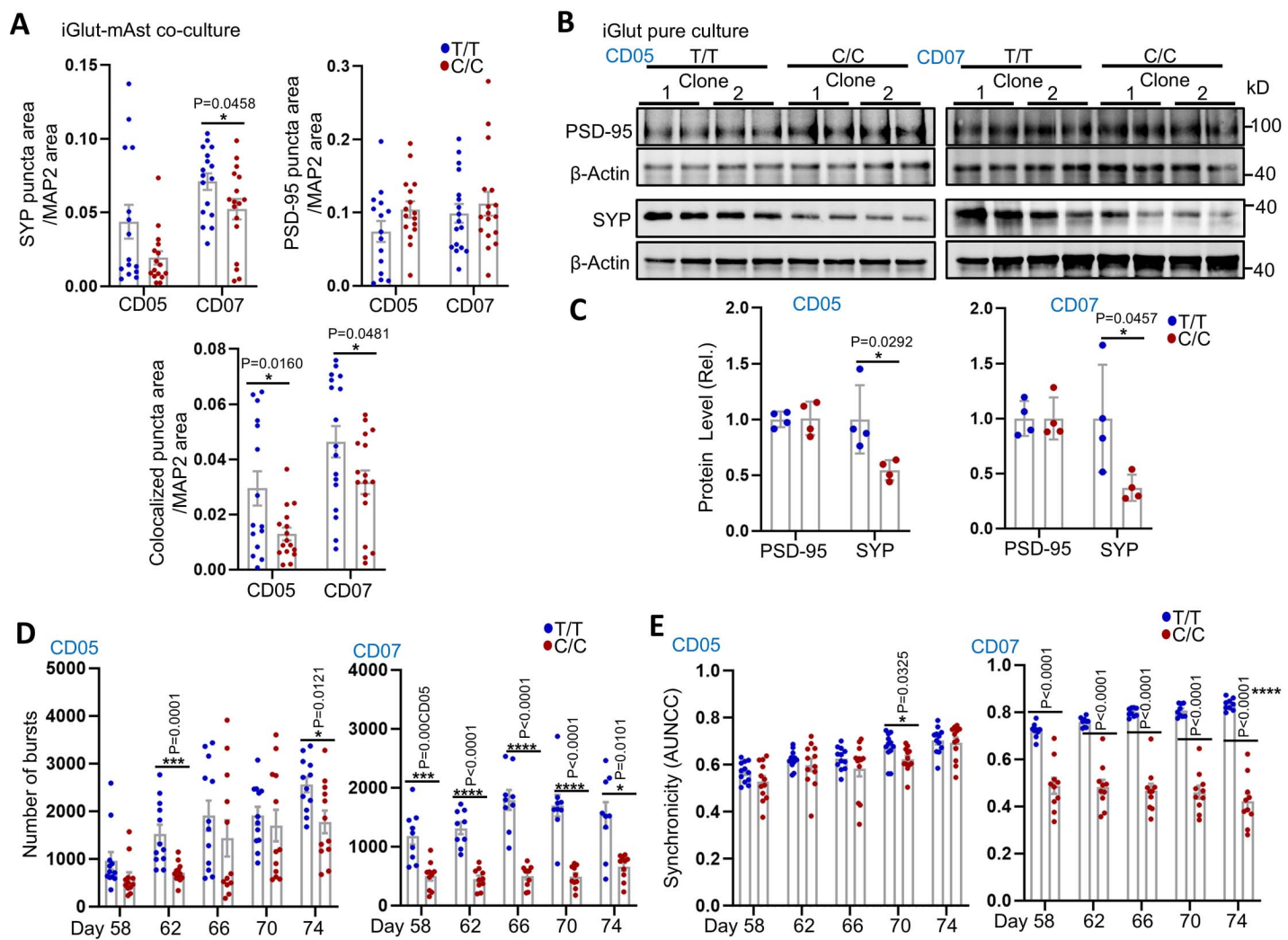

Supplement: Supplementary file 1 — Additional file 1. Figure S1: Bioinformatic and experimental validation of the regulatory effect of rs1532278 on TF-binding and CLU expression, related to Fig. 1. (A) Multiz alignment and phyloP conservation (470 mammals) around rs1532278 (from UCSC hg38 genome browser). (B) JASPAR predicted TF binding sites at rs1532278 and TF expression levels in iGlut (10/23 predicted TFs can be detected by RNA seq). CPM, counts per million reads. (C) Representative images (CD07 line) of iGlut of all three genotypes are also shown, related to Fig. 1D (bottom panel); GFAP and HuNu (human nuclear antigen) staining shows specificity of HuNu and MAP2 staining for iGlut in iGlut-mAst co-cultures. (D) No difference of iGlut differentiation efficiency were found between T/T and CC carriers in iGlut-mAst co-cultures, and none proliferating cells were observed in these neurons indicated by negative staining of Ki67. HuNu +, human cells. n=5 coverslips per group from one differentiation of both CD05 and CD07 lines (2-3 clones per line, one coverslip per clone and 4-5 images per coverslip). (E) DRGX ChIP-qPCR for iGlut-mAst co-cultures of CD07 line on day 30. n=3 biological replicates per group (one clone with 3 biological replicates from the CD07 line) from one independent differentiation. (F-G) ISL2 siRNA knockdown in day-30 pure iGlut (C/C) cultures. Samples of 72 hours post-siRNA transfection were used for qPCR. n=3 biological replicates from one clone per line in one independent differentiation. (H) CLU mRNA levels in iGlut pure cultures. n=6 biological replicates per group (2-3 clones per line and 2-3 biological replicates for each clone) from two independent differentiations of each line (I) sCLU levels detected by ELISA from the supernatant of iGlut pure cultures. n=4 biological replicates per group (2 clones per line and 2 biological replicates for each clone) from two independent differentiations of each line. (J) CLU mRNA levels of mAst in iGlut-mAst co-cultures. n=4 biological r [file 13024_2025_840_MOESM1_ESM.zip › figure S3.pdf]

**A**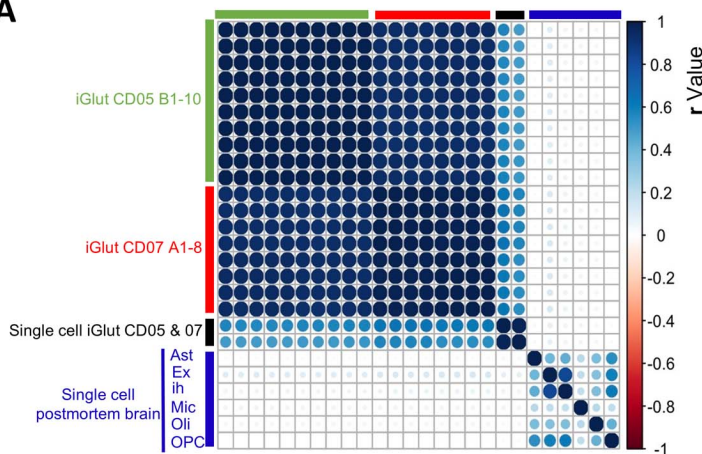**B**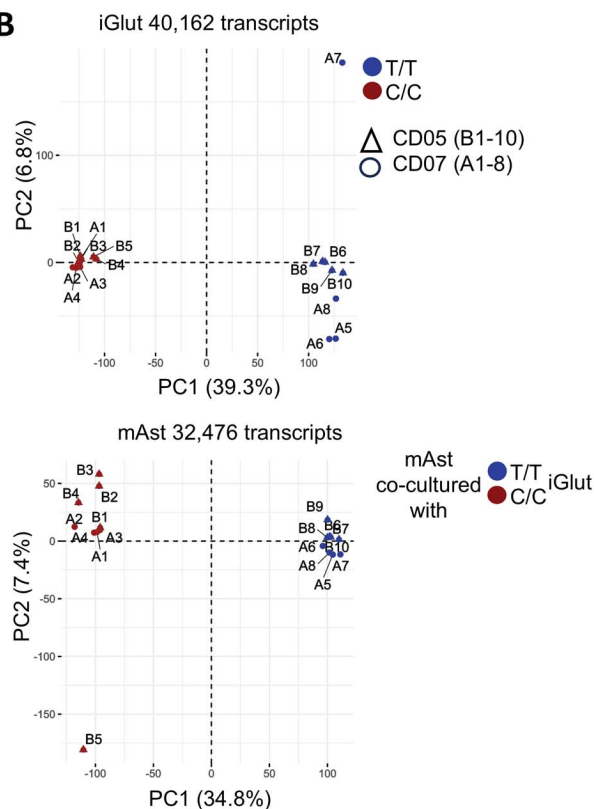**C**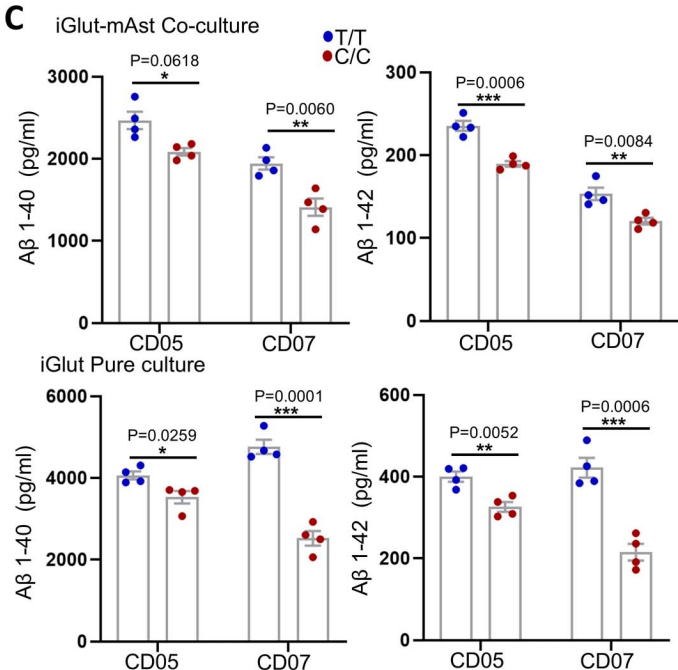**D**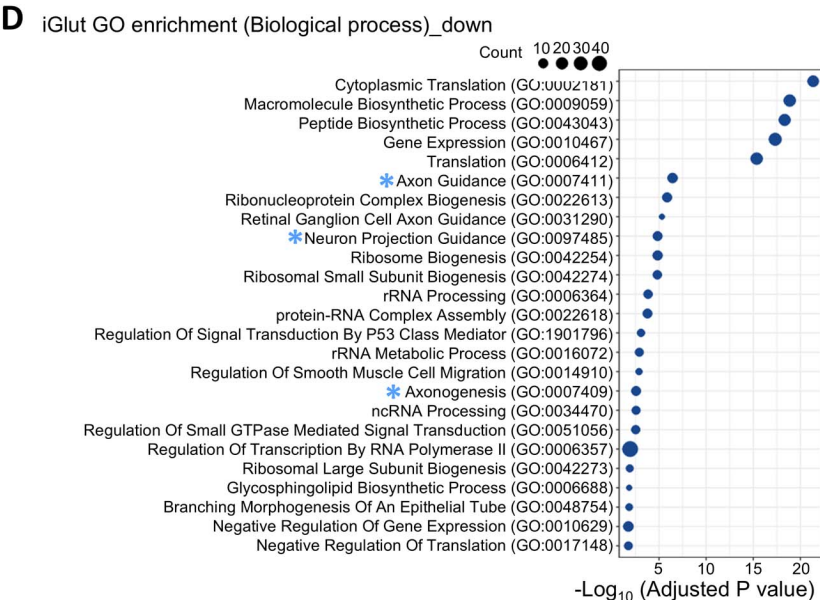**E**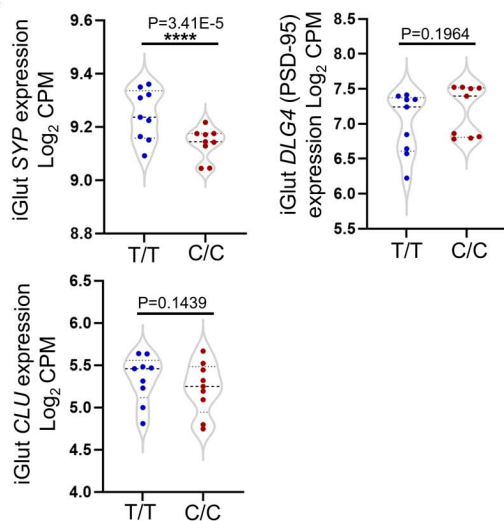**F**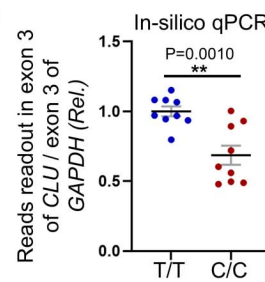

Supplement: Supplementary file 1 — Additional file 1. Figure S1: Bioinformatic and experimental validation of the regulatory effect of rs1532278 on TF-binding and CLU expression, related to Fig. 1. (A) Multiz alignment and phyloP conservation (470 mammals) around rs1532278 (from UCSC hg38 genome browser). (B) JASPAR predicted TF binding sites at rs1532278 and TF expression levels in iGlut (10/23 predicted TFs can be detected by RNA seq). CPM, counts per million reads. (C) Representative images (CD07 line) of iGlut of all three genotypes are also shown, related to Fig. 1D (bottom panel); GFAP and HuNu (human nuclear antigen) staining shows specificity of HuNu and MAP2 staining for iGlut in iGlut-mAst co-cultures. (D) No difference of iGlut differentiation efficiency were found between T/T and CC carriers in iGlut-mAst co-cultures, and none proliferating cells were observed in these neurons indicated by negative staining of Ki67. HuNu +, human cells. n=5 coverslips per group from one differentiation of both CD05 and CD07 lines (2-3 clones per line, one coverslip per clone and 4-5 images per coverslip). (E) DRGX ChIP-qPCR for iGlut-mAst co-cultures of CD07 line on day 30. n=3 biological replicates per group (one clone with 3 biological replicates from the CD07 line) from one independent differentiation. (F-G) ISL2 siRNA knockdown in day-30 pure iGlut (C/C) cultures. Samples of 72 hours post-siRNA transfection were used for qPCR. n=3 biological replicates from one clone per line in one independent differentiation. (H) CLU mRNA levels in iGlut pure cultures. n=6 biological replicates per group (2-3 clones per line and 2-3 biological replicates for each clone) from two independent differentiations of each line (I) sCLU levels detected by ELISA from the supernatant of iGlut pure cultures. n=4 biological replicates per group (2 clones per line and 2 biological replicates for each clone) from two independent differentiations of each line. (J) CLU mRNA levels of mAst in iGlut-mAst co-cultures. n=4 biological r [file 13024_2025_840_MOESM1_ESM.zip › figure S5.pdf]

**A**

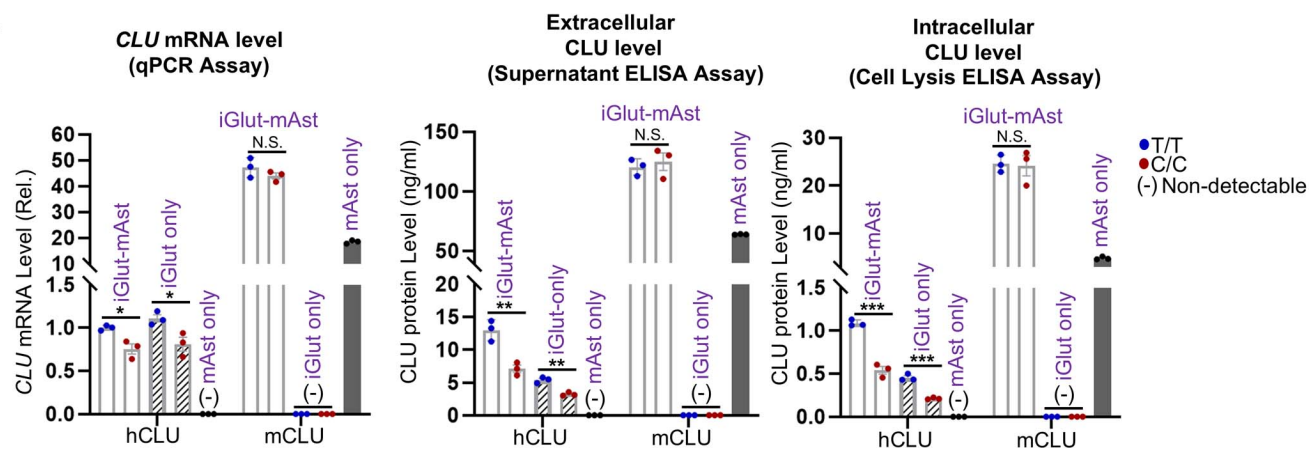

**B**

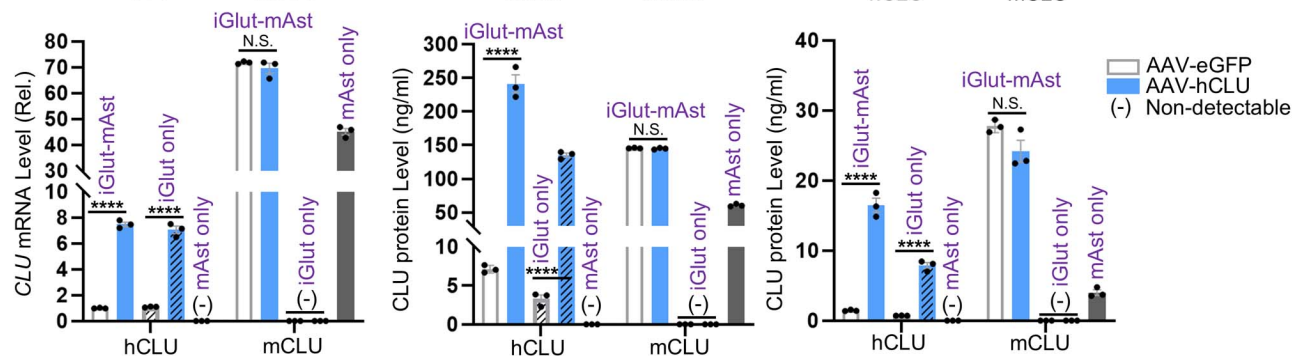

**C**

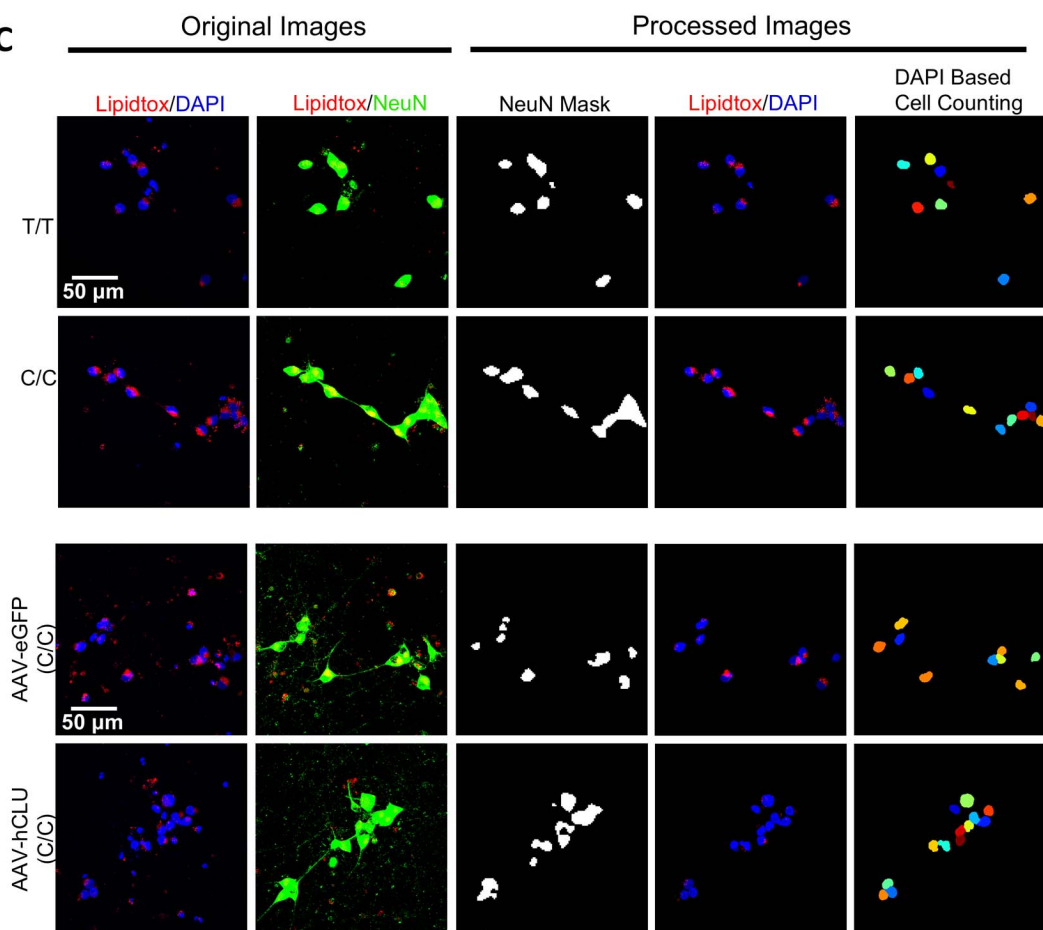

Supplement: Supplementary file 1 — Additional file 1. Figure S1: Bioinformatic and experimental validation of the regulatory effect of rs1532278 on TF-binding and CLU expression, related to Fig. 1. (A) Multiz alignment and phyloP conservation (470 mammals) around rs1532278 (from UCSC hg38 genome browser). (B) JASPAR predicted TF binding sites at rs1532278 and TF expression levels in iGlut (10/23 predicted TFs can be detected by RNA seq). CPM, counts per million reads. (C) Representative images (CD07 line) of iGlut of all three genotypes are also shown, related to Fig. 1D (bottom panel); GFAP and HuNu (human nuclear antigen) staining shows specificity of HuNu and MAP2 staining for iGlut in iGlut-mAst co-cultures. (D) No difference of iGlut differentiation efficiency were found between T/T and CC carriers in iGlut-mAst co-cultures, and none proliferating cells were observed in these neurons indicated by negative staining of Ki67. HuNu +, human cells. n=5 coverslips per group from one differentiation of both CD05 and CD07 lines (2-3 clones per line, one coverslip per clone and 4-5 images per coverslip). (E) DRGX ChIP-qPCR for iGlut-mAst co-cultures of CD07 line on day 30. n=3 biological replicates per group (one clone with 3 biological replicates from the CD07 line) from one independent differentiation. (F-G) ISL2 siRNA knockdown in day-30 pure iGlut (C/C) cultures. Samples of 72 hours post-siRNA transfection were used for qPCR. n=3 biological replicates from one clone per line in one independent differentiation. (H) CLU mRNA levels in iGlut pure cultures. n=6 biological replicates per group (2-3 clones per line and 2-3 biological replicates for each clone) from two independent differentiations of each line (I) sCLU levels detected by ELISA from the supernatant of iGlut pure cultures. n=4 biological replicates per group (2 clones per line and 2 biological replicates for each clone) from two independent differentiations of each line. (J) CLU mRNA levels of mAst in iGlut-mAst co-cultures. n=4 biological r [file 13024_2025_840_MOESM1_ESM.zip › Figure S6.pdf]

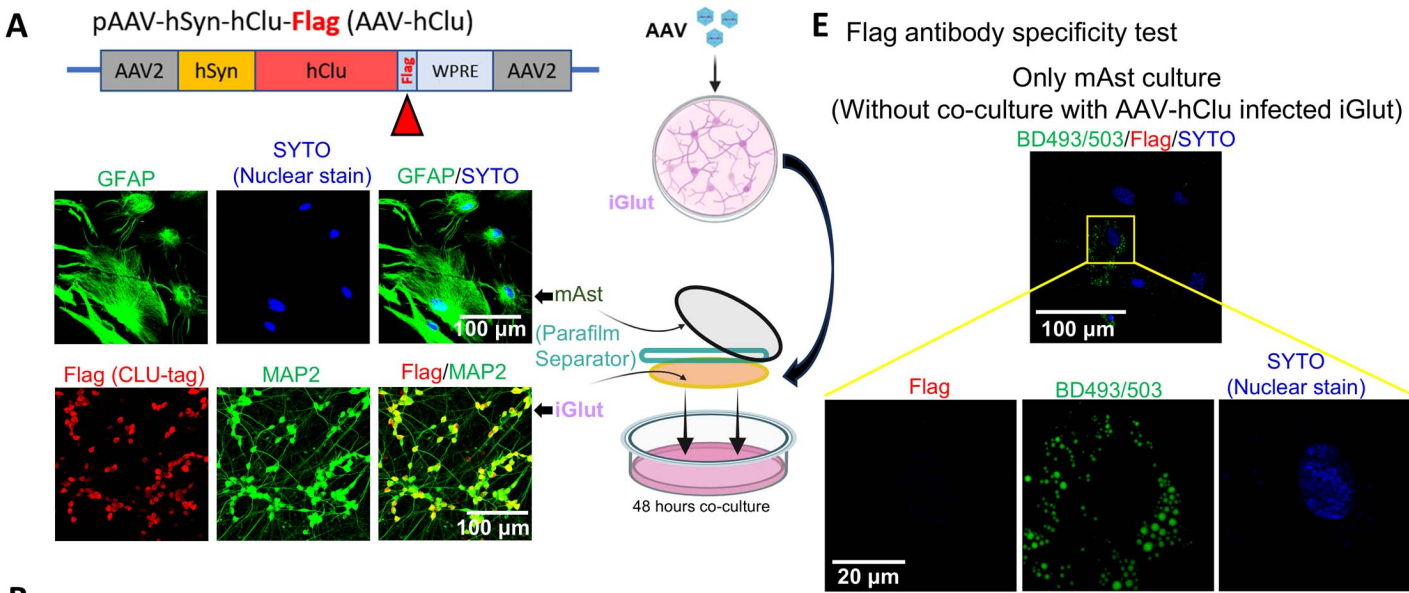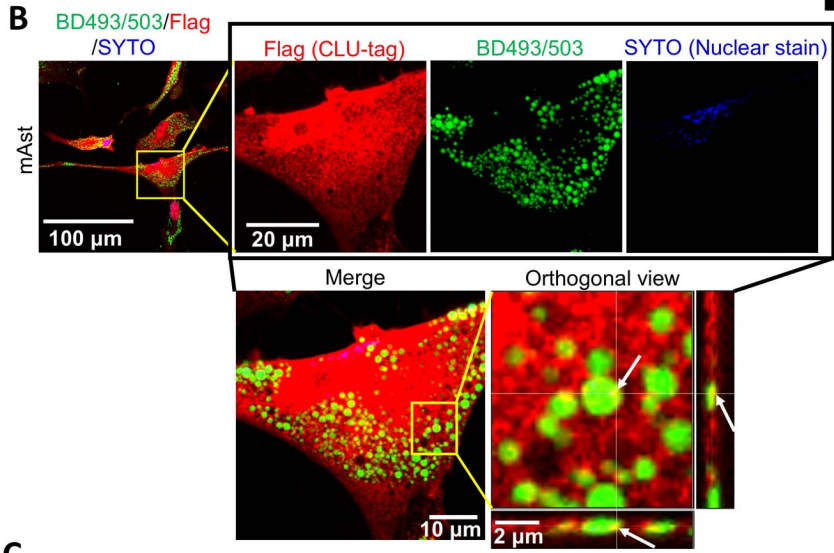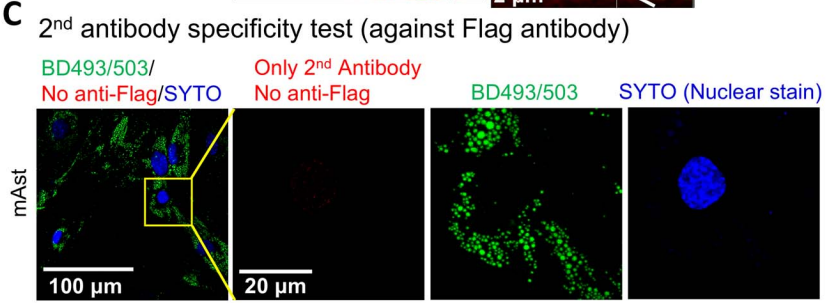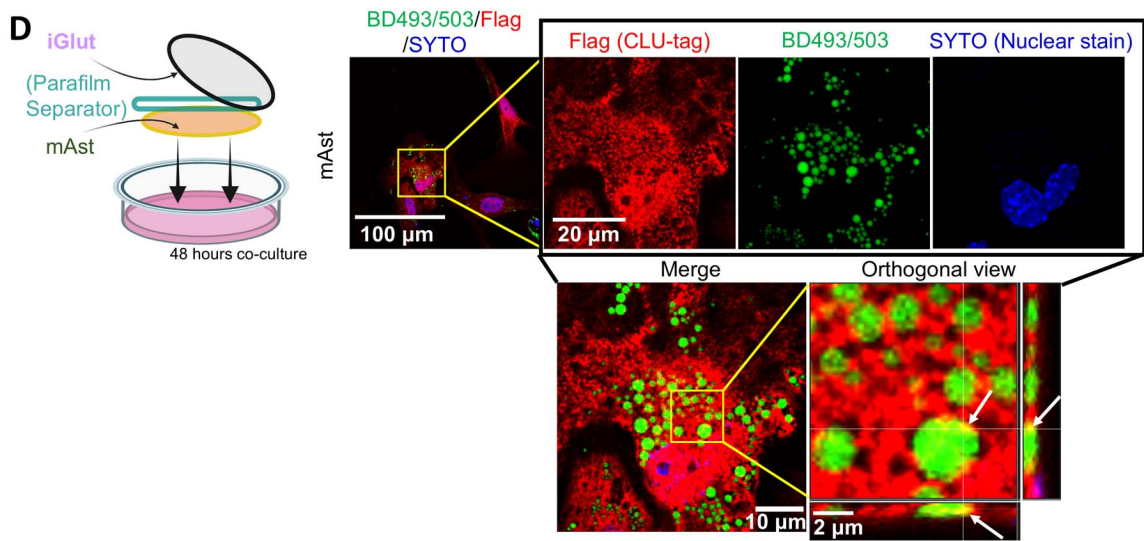

Supplement: Supplementary file 1 — Additional file 1. Figure S1: Bioinformatic and experimental validation of the regulatory effect of rs1532278 on TF-binding and CLU expression, related to Fig. 1. (A) Multiz alignment and phyloP conservation (470 mammals) around rs1532278 (from UCSC hg38 genome browser). (B) JASPAR predicted TF binding sites at rs1532278 and TF expression levels in iGlut (10/23 predicted TFs can be detected by RNA seq). CPM, counts per million reads. (C) Representative images (CD07 line) of iGlut of all three genotypes are also shown, related to Fig. 1D (bottom panel); GFAP and HuNu (human nuclear antigen) staining shows specificity of HuNu and MAP2 staining for iGlut in iGlut-mAst co-cultures. (D) No difference of iGlut differentiation efficiency were found between T/T and CC carriers in iGlut-mAst co-cultures, and none proliferating cells were observed in these neurons indicated by negative staining of Ki67. HuNu +, human cells. n=5 coverslips per group from one differentiation of both CD05 and CD07 lines (2-3 clones per line, one coverslip per clone and 4-5 images per coverslip). (E) DRGX ChIP-qPCR for iGlut-mAst co-cultures of CD07 line on day 30. n=3 biological replicates per group (one clone with 3 biological replicates from the CD07 line) from one independent differentiation. (F-G) ISL2 siRNA knockdown in day-30 pure iGlut (C/C) cultures. Samples of 72 hours post-siRNA transfection were used for qPCR. n=3 biological replicates from one clone per line in one independent differentiation. (H) CLU mRNA levels in iGlut pure cultures. n=6 biological replicates per group (2-3 clones per line and 2-3 biological replicates for each clone) from two independent differentiations of each line (I) sCLU levels detected by ELISA from the supernatant of iGlut pure cultures. n=4 biological replicates per group (2 clones per line and 2 biological replicates for each clone) from two independent differentiations of each line. (J) CLU mRNA levels of mAst in iGlut-mAst co-cultures. n=4 biological r [file 13024_2025_840_MOESM1_ESM.zip › Figure S7.pdf]

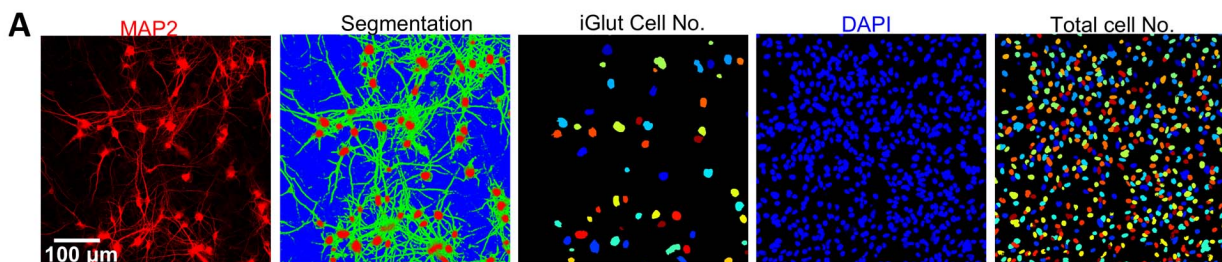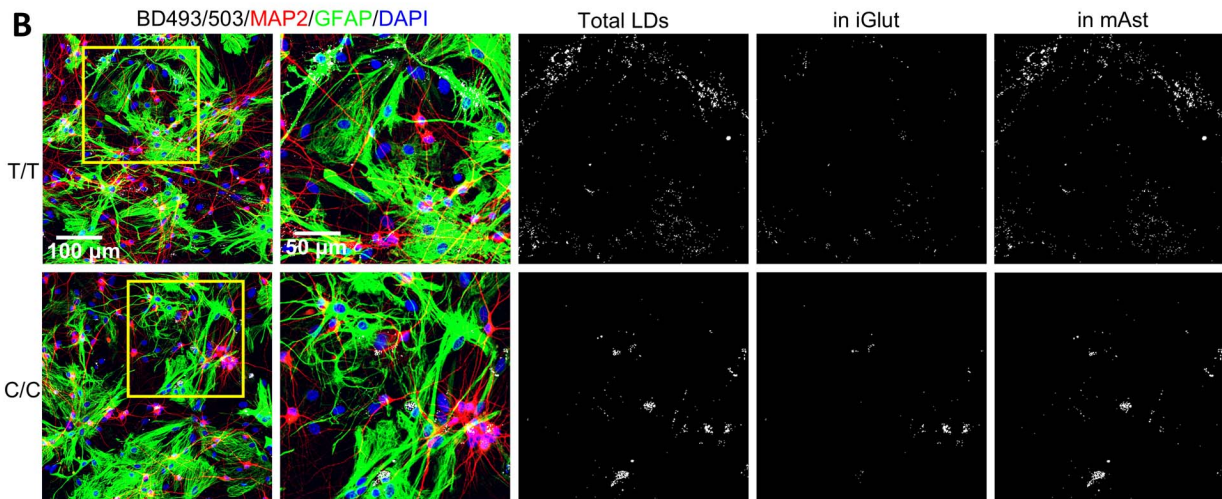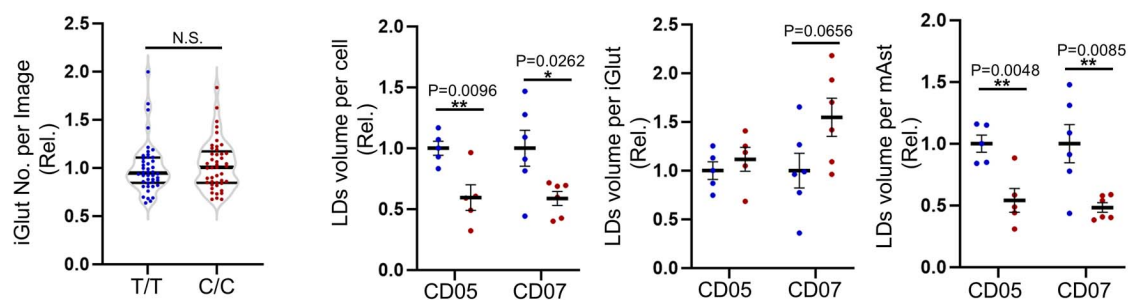

Supplement: Supplementary file 1 — Additional file 1. Figure S1: Bioinformatic and experimental validation of the regulatory effect of rs1532278 on TF-binding and CLU expression, related to Fig. 1. (A) Multiz alignment and phyloP conservation (470 mammals) around rs1532278 (from UCSC hg38 genome browser). (B) JASPAR predicted TF binding sites at rs1532278 and TF expression levels in iGlut (10/23 predicted TFs can be detected by RNA seq). CPM, counts per million reads. (C) Representative images (CD07 line) of iGlut of all three genotypes are also shown, related to Fig. 1D (bottom panel); GFAP and HuNu (human nuclear antigen) staining shows specificity of HuNu and MAP2 staining for iGlut in iGlut-mAst co-cultures. (D) No difference of iGlut differentiation efficiency were found between T/T and CC carriers in iGlut-mAst co-cultures, and none proliferating cells were observed in these neurons indicated by negative staining of Ki67. HuNu +, human cells. n=5 coverslips per group from one differentiation of both CD05 and CD07 lines (2-3 clones per line, one coverslip per clone and 4-5 images per coverslip). (E) DRGX ChIP-qPCR for iGlut-mAst co-cultures of CD07 line on day 30. n=3 biological replicates per group (one clone with 3 biological replicates from the CD07 line) from one independent differentiation. (F-G) ISL2 siRNA knockdown in day-30 pure iGlut (C/C) cultures. Samples of 72 hours post-siRNA transfection were used for qPCR. n=3 biological replicates from one clone per line in one independent differentiation. (H) CLU mRNA levels in iGlut pure cultures. n=6 biological replicates per group (2-3 clones per line and 2-3 biological replicates for each clone) from two independent differentiations of each line (I) sCLU levels detected by ELISA from the supernatant of iGlut pure cultures. n=4 biological replicates per group (2 clones per line and 2 biological replicates for each clone) from two independent differentiations of each line. (J) CLU mRNA levels of mAst in iGlut-mAst co-cultures. n=4 biological r [file 13024_2025_840_MOESM1_ESM.zip › figure S8.pdf]

**A**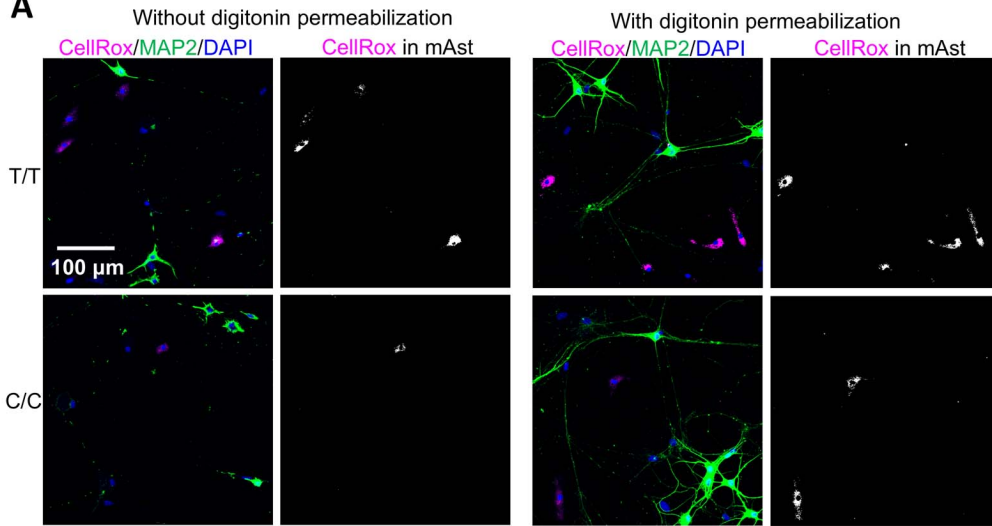**B**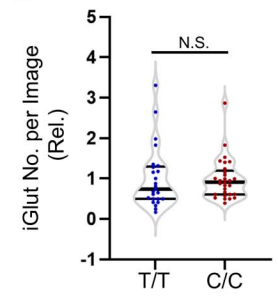**C**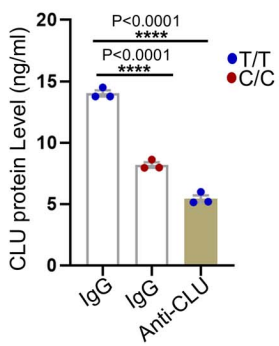**D** From mAst Upregulated gene sets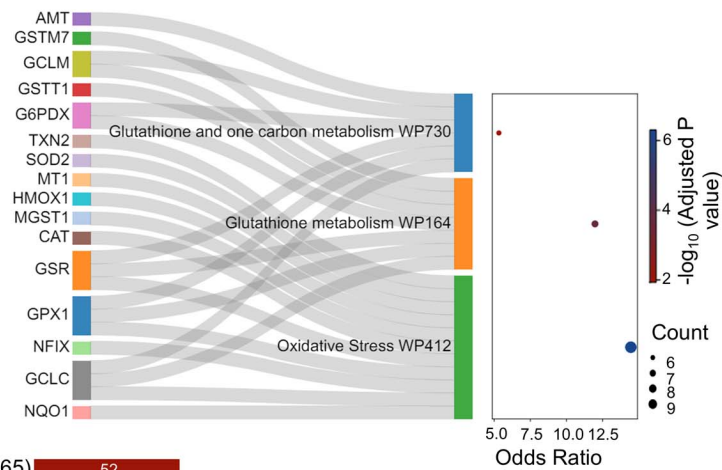**E**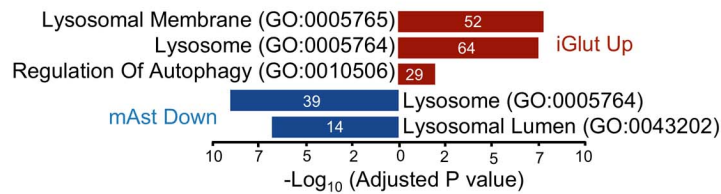

Supplement: Supplementary file 1 — Additional file 1. Figure S1: Bioinformatic and experimental validation of the regulatory effect of rs1532278 on TF-binding and CLU expression, related to Fig. 1. (A) Multiz alignment and phyloP conservation (470 mammals) around rs1532278 (from UCSC hg38 genome browser). (B) JASPAR predicted TF binding sites at rs1532278 and TF expression levels in iGlut (10/23 predicted TFs can be detected by RNA seq). CPM, counts per million reads. (C) Representative images (CD07 line) of iGlut of all three genotypes are also shown, related to Fig. 1D (bottom panel); GFAP and HuNu (human nuclear antigen) staining shows specificity of HuNu and MAP2 staining for iGlut in iGlut-mAst co-cultures. (D) No difference of iGlut differentiation efficiency were found between T/T and CC carriers in iGlut-mAst co-cultures, and none proliferating cells were observed in these neurons indicated by negative staining of Ki67. HuNu +, human cells. n=5 coverslips per group from one differentiation of both CD05 and CD07 lines (2-3 clones per line, one coverslip per clone and 4-5 images per coverslip). (E) DRGX ChIP-qPCR for iGlut-mAst co-cultures of CD07 line on day 30. n=3 biological replicates per group (one clone with 3 biological replicates from the CD07 line) from one independent differentiation. (F-G) ISL2 siRNA knockdown in day-30 pure iGlut (C/C) cultures. Samples of 72 hours post-siRNA transfection were used for qPCR. n=3 biological replicates from one clone per line in one independent differentiation. (H) CLU mRNA levels in iGlut pure cultures. n=6 biological replicates per group (2-3 clones per line and 2-3 biological replicates for each clone) from two independent differentiations of each line (I) sCLU levels detected by ELISA from the supernatant of iGlut pure cultures. n=4 biological replicates per group (2 clones per line and 2 biological replicates for each clone) from two independent differentiations of each line. (J) CLU mRNA levels of mAst in iGlut-mAst co-cultures. n=4 biological r [file 13024_2025_840_MOESM1_ESM.zip › figure S9.pdf]
